# An End-to-End Fault Diagnosis Model for Rolling Bearings Based on Multi-Scale Convolution and the Kolmogorov–Arnold Network

**DOI:** 10.3390/s26134005

**Published:** 2026-06-24

**Authors:** Donghua Yu, Zhenyu Wang, Jia Liu, Huan Liu, Changtian Ying

**Affiliations:** Department of Computer Science and Engineering, Shaoxing University, No. 508 Huancheng West Road, Shaoxing 312000, China; donghuayu@usx.edu.cn (D.Y.); 24020855142@usx.edu.cn (Z.W.); 25020854109@usx.edu.cn (J.L.); 25020854118@usx.edu.cn (H.L.)

**Keywords:** rolling bearing, fault diagnosis, multi-scale convolution, SE attention mechanism, Kolmogorov–Arnold Network, vibration signal

## Abstract

Rolling bearings, as core components of rotating machinery, are prone to failure under harsh working conditions, and their fault diagnosis is crucial for the safe operation of industrial systems. Aiming at resolving the problems of weak fault feature representation, poor model generalization ability and high dependence on manual preprocessing in traditional bearing fault diagnosis methods, an end-to-end fault diagnosis model named KanMSConv is proposed for one-dimensional raw vibration signals. The model abandons complex time–frequency transformation and manual feature engineering, and constructs a multi-scale feature extraction module based on depthwise separable convolution to capture local impulsive components and global modulation characteristics of fault signals simultaneously. The SE channel attention mechanism is integrated to adaptively enhance fault-related critical features and reduce redundant channel responses. Residual connection is introduced to alleviate the gradient degradation problem of deep networks and improve feature reuse capability. On this basis, the Kolmogorov–Arnold Network (KAN) is used to replace the traditional fully connected layer, which enhances the model’s ability to fit complex nonlinear mapping relationships and distinguish fault classification boundaries. Experimental verification is carried out on three representative rolling bearing datasets (CWRU, PU, SDUST) under multi-load, multi-class and cross-platform conditions. The results show that the KanMSConv model achieves 100% accuracy on the CWRU dataset, 99.93% on the PU dataset and 99.80% on the SDUST dataset, which is significantly superior to the existing mainstream fault diagnosis models in terms of Accuracy, Precision, Recall and F1-Score. And the ablation and computational cost analyses further support this conclusion.

## 1. Introduction

In modern industrial systems, rotating machinery is extensively employed in key sectors such as manufacturing, transportation, energy and power, and heavy equipment. Its operational condition directly influences equipment efficiency, service life, and overall system safety. As one of the most critical fundamental components in rotating machinery, rolling bearings are responsible for load support and power transmission, and they often operate under high-speed, heavy-load, impact, and complex coupled working conditions. Under such harsh service environments, rolling bearings are highly vulnerable to defects such as wear, cracks, spalling, and fatigue failure. Since bearing faults are typically characterized by weak incipient signatures, concealed degradation processes, and vibration responses that are easily contaminated by environmental noise, failure to detect them in a timely manner may not only degrade equipment performance, intensify vibration, and increase maintenance costs, but may also lead to unexpected shutdowns and even serious safety accidents [1]. Therefore, achieving efficient, accurate, and robust fault identification for rolling bearings under complex operating conditions and noisy backgrounds has become a critical issue in the fields of intelligent operation and maintenance as well as industrial fault diagnosis. Research on this topic is of great theoretical significance and practical engineering value for improving equipment reliability, ensuring production safety, reducing unplanned downtime losses, and promoting the implementation of condition monitoring and predictive maintenance technologies [2].

Traditional rolling bearing fault diagnosis methods mainly rely on signal processing techniques, including time-domain statistical analysis, frequency-domain analysis, envelope demodulation, and time–frequency transformation, and then combine these handcrafted features with shallow classifiers such as support vector machines and random forests to accomplish fault identification. Although these methods exhibit a certain degree of physical interpretability, they are generally dependent on expert knowledge for feature construction and parameter selection. In practical scenarios involving strong noise interference, significant working condition fluctuations, and complex sample distributions, such methods often struggle to simultaneously achieve satisfactory feature representation capability, diagnostic accuracy, and generalization performance [3]. With the rapid development of intelligent manufacturing and industrial monitoring technologies, deep learning has gradually emerged as an important research paradigm in rolling bearing fault diagnosis. Owing to its end-to-end feature learning capability, deep learning can automatically extract hierarchical fault representations directly from raw signals, thereby significantly reducing the reliance on manual feature engineering.

In recent years, a large number of studies have focused on optimizing deep learning models for rolling bearing fault diagnosis in challenging scenarios, such as small-sample conditions, strong noise interference, varying operating conditions, and cross-platform tasks, and considerable progress has been achieved. To address the difficulty of identifying weak fault signatures under complex noise backgrounds, one-dimensional convolutional neural networks have been widely adopted due to their ability to directly model raw vibration signals in an end-to-end manner, showing strong potential in fault feature extraction and classification [4,5]. To capture fault patterns distributed over different temporal scales, multi-scale convolutional structures have been extensively introduced to jointly model local impulsive information and global modulation characteristics [6]. In addition, attention mechanisms have been incorporated to enhance the adaptive selection of discriminative fault-related features, thereby further improving the model’s diagnostic performance [7]. Meanwhile, transfer learning and domain adaptation methods have provided new perspectives for improving diagnostic generalization under cross-condition and cross-platform scenarios [8].

Benchmark accuracy and industrial validity are related but distinct questions. Controlled test rig datasets enable reproducible method comparison, but their class definitions, acquisition conditions, and partition protocols can make classification substantially easier than diagnosis on unseen industrial machines. Benchmark studies have therefore emphasized careful interpretation of reported accuracy and transparent evaluation protocols [9,10]. In this study, the terms “multi-load” and “cross-platform” describe evaluation across the selected laboratory datasets and operating settings; they do not imply field validation under uncontrolled industrial interference.

Despite the substantial progress achieved in fault feature extraction, model design, and complex-scenario adaptation, several issues remain insufficiently addressed. First, some existing methods heavily depend on time–frequency transformation, image encoding, signal decomposition, or multi-stage preprocessing procedures. Although these strategies can enhance fault feature representation to a certain extent, they inevitably increase the complexity of model implementation and the cost of online deployment, which limits their applicability in real-time industrial environments. Second, many high-performance diagnostic models rely on multi-branch architectures, multi-module stacking, or sophisticated feature fusion schemes. While these designs improve representation capability, they also introduce large parameter scales, high computational burden, and increased training complexity. Third, in multi-condition, cross-platform, and even cross-dataset scenarios, vibration signals usually exhibit strong non-stationarity, significant distribution shift, and insufficient feature consistency, making it difficult for existing methods to maintain robust stability and satisfactory generalization performance. Finally, the KAN network [11], as a recently proposed novel neural architecture, has demonstrated promising potential in terms of representation ability and interpretability. However, current studies on KAN mainly focus on general machine learning tasks, while its application to one-dimensional raw vibration signals for rolling bearing fault diagnosis remains relatively limited. Therefore, it is meaningful to evaluate whether a compact framework combining multi-scale convolution and KAN can improve classification on established bearing benchmarks while clearly defining the limits of laboratory evidence.

**Figure 1:**
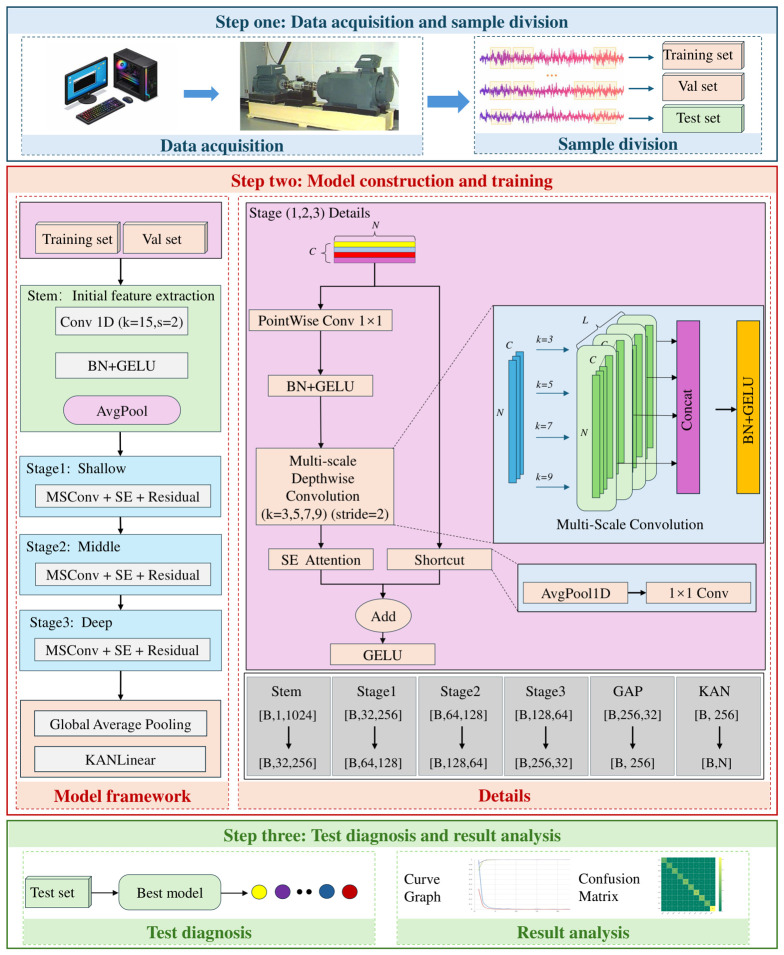
KanMSConv model framework diagram.

To address the above issues, this paper proposes an end-to-end fault diagnosis model, termed KanMSConv, for one-dimensional raw vibration signals. The main contributions are summarized as follows:

1. An end-to-end fault diagnosis framework, KanMSConv, is proposed based on the direct input of one-dimensional raw vibration signals, as shown in Figure 1, thereby avoiding reliance on complex time–frequency transformations and manual feature engineering.
2. A parallel multi-scale depthwise separable convolution module integrated with the SE channel attention mechanism is constructed. The module is designed to model impulse components, periodic modulation information, and local details from different receptive fields while recalibrating fault-related channel responses.
3. A KAN module is introduced to replace the traditional fully connected layer, so as to enhance the model’s ability to represent complex nonlinear mapping relationships and class decision boundaries.
4. Experimental results on three representative bearing datasets, namely CWRU, PU, and SDUST, under multi-load, multi-class, and multi-platform conditions demonstrate that the proposed KanMSConv model outperforms the comparative models in terms of Accuracy, Precision, Recall, and F1-Score. And the ablation and computational cost analyses further support this conclusion.

## 2. Related Work

### 2.1. Classic Diagnostic Methods for Bearing Failure

Traditional rolling bearing fault diagnosis methods mainly rely on handcrafted feature extraction and shallow classification models. In general, fault-related information is extracted through time-domain statistical indicators, envelope spectrum analysis, frequency-domain peak features, and time–frequency representations, and then combined with classifiers such as support vector machines, k-nearest neighbors, or random forests for condition identification. Although these methods offer a certain degree of interpretability under specific operating conditions, they are highly dependent on expert experience. Moreover, under complex noise interference, fluctuating working conditions, and limited sample availability, manually constructed features often fail to consistently capture the essential characteristics of faults. Smith and Randall systematically examined the CWRU bearing data using established diagnostic techniques and emphasized that benchmark protocols and dataset characteristics must be considered when interpreting algorithmic performance [9].

With the development of deep learning, fault diagnosis research has gradually shifted from handcrafted feature-driven approaches to data-driven automatic representation learning. Existing studies have shown that convolutional neural networks can directly extract hierarchical features from raw vibration signals, significantly reducing the complexity of traditional feature engineering and achieving better diagnostic performance than shallow methods in rolling bearing fault diagnosis. For example, Janssens et al. proposed CNN-based feature learning for rotating machinery fault detection to reduce reliance on manually engineered vibration features [12]. Eren directly applied a one-dimensional CNN to bearing fault signals, while Ince et al. developed a compact 1-D CNN for real-time motor fault detection [4,5]. However, when dealing with small-sample, imbalanced-sample, and high-noise scenarios, conventional deep networks still tend to suffer from unstable training, feature suppression, and blurred decision boundaries. To address these issues, ref. [13] proposed a data augmentation method based on compressed sensing, which reconstructs limited fault samples to expand the training data distribution, thereby alleviating the training instability caused by the scarcity of labeled samples in industrial scenarios and enabling deep models to maintain satisfactory recognition performance under small-sample conditions. Ref. [14] developed a diagnostic method combining CEEMDAN and CNN-SVM, in which signal decomposition is first employed to suppress noise and modal aliasing, followed by deep convolutional feature extraction and classifier-based discrimination. This strategy improves the problems of feature suppression and blurred boundaries encountered by conventional deep networks in high-noise environments. Ref. [15] further integrated a denoising diffusion implicit model with TransNet, using generative augmentation to mitigate class imbalance and then employing a deep network for fault classification. Such studies indicate that, in imbalanced-sample and weak-fault scenarios, the joint optimization of data generation and feature learning has become an important avenue for improving diagnostic robustness.

### 2.2. Research on Multi-Scale Feature Extraction and Temporal Modeling

Rolling bearing fault vibration signals usually contain transient impulsive components, periodic modulation patterns, and long-range dependency information simultaneously. As a result, convolutional structures with a single receptive field often struggle to achieve joint representation of fault features across different scales. Consequently, multi-scale modeling has gradually become one of the major directions in current fault diagnosis research. Ref. [16] proposed a method based on the Gramian Angular Difference Field and an improved dual-attention residual network. By transforming one-dimensional vibration signals into image representations and incorporating a dual-attention mechanism, their method enhanced the model’s sensitivity to weak fault features. Ref. [17] developed a multi-branch selectively fused deep residual network, in which differentiated extraction and fusion strategies were designed for input features from different sources, thereby improving the utilization efficiency of multi-source heterogeneous information. In 2025, ref. [18] proposed MR-FuSN, which further filtered and integrated features at different scales from the perspective of multi-resolution fusion, thereby strengthening the multi-scale representation capability under complex noisy backgrounds.

In addition to multi-scale convolution, temporal dependency modeling is also an important means of improving fault identification performance. Ref. [19] proposed a CNN-LSTM-GRU hybrid model, in which convolution was first used to extract local patterns, followed by LSTM and GRU to jointly model the long-term and short-term dependency relationships in the fault evolution process, thus enhancing the model’s ability to characterize dynamically changing features. Ref. [20] introduced a method combining CNN-BiLSTM with residual modules, where bidirectional temporal modeling was employed to improve the contextual representation of fault features. Ref. [21] further proposed a hybrid framework integrating denoising multi-view CNN, Mamba, and enhanced adaptive self-attention LSTM, which simultaneously captured local details and long-range dependencies under strong noise and long-sequence conditions, demonstrating the advantages of collaborative modeling between temporal networks and attention mechanisms. These studies indicate that multi-scale modeling and temporal dependency modeling have become important approaches for improving fault diagnosis performance under complex operating conditions. However, some of these methods still rely on image-based representations or complex network stacking, resulting in relatively heavy model structures.

### 2.3. Research on Transfer Learning, Domain Adaptation and Cross-Condition Diagnosis

In practical industrial scenarios, variations in operating load, rotational speed, installation environment, and sampling conditions often lead to significant distribution discrepancies between source-domain and target-domain data. As a result, models trained under a single operating condition tend to suffer performance degradation when applied to cross-condition, cross-equipment, and cross-platform tasks. To alleviate this issue, transfer learning and domain adaptation methods have been widely employed to improve model generalization capability. Ref. [22] proposed a deep reconstruction transfer convolutional neural network, which enhances diagnostic performance under inconsistent source-domain and target-domain distributions through the collaborative optimization of reconstruction learning and cross-domain feature transfer. Ref. [23] developed a diagnostic method based on an adaptive residual shrinkage network and transfer learning, which improved target-domain adaptation performance in scenarios where limited samples and varying operating conditions coexist. Ref. [24] further proposed a domain-adversarial transfer learning model incorporating a structural adjustment module, which reduces inter-domain discrepancies through adversarial training and enhances feature stability under varying working conditions. Ref. [25] combined synchrosqueezed wavelet transform with a transfer residual convolutional network, thereby improving cross-domain recognition capability on the basis of strengthened time–frequency feature representation.

On this basis, researchers have further improved model design from the perspectives of finer-grained distribution matching and cross-domain feature organization. Ref. [26] integrated a multi-scale residual network with MK-MMD to enhance the consistency of feature representations across different domains and mitigate the distribution shift caused by operating condition variation. Ref. [27] proposed a distribution alignment metric based on variance difference representation, enabling the model to focus more effectively on stable features closely related to fault categories. Ref. [28] introduced an unsupervised transfer learning method based on SOCNN and FBNN, which strengthened the applicability of the model in the absence of target-domain labels. Ref. [29] proposed MAJATNet, which embeds joint maximum mean discrepancy and adversarial loss into a lightweight multi-scale attention framework, achieving a good balance among cross-domain performance, network size, and training stability. These studies indicate that cross-domain adaptation has become a critical issue in intelligent fault diagnosis; however, most existing methods still rely on additional alignment strategies or relatively complex training mechanisms.

### 2.4. Diagnostic Methods Related to KAN Network Modules

In recent years, KAN networks have attracted increasing attention due to their idea of replacing traditional linear weight connections with learnable univariate functions, thereby demonstrating strong potential for nonlinear modeling. Accordingly, they have also begun to be introduced into the field of fault diagnosis. Ref. [30] proposed the KANs-CNN method, in which KAN was employed to enhance nonlinear representation capability in small-sample fault diagnosis, thereby improving the model’s ability to capture complex time–frequency features. Ref. [31] developed the KAN-HyperMP model, which combines hypergraph message passing with KANLinear for rolling bearing fault identification under noisy environments, thus improving complex relationship modeling and anti-noise capability. Ref. [32] proposed an automated port-bearing fault detection framework based on time–frequency filtering and CNN-KAN, demonstrating the application potential of combining KAN with convolutional structures in engineering equipment diagnosis. Ref. [33] further introduced the GraphKAN diagnostic method, in which features extracted by CNN-KAN were further organized into a graph structure for cross-domain discrimination, thereby enhancing the modeling capability of feature correlations under variable operating conditions. Ref. [34] proposed KANConv-ACGCA-SENet, which integrates KAN convolution, dual-branch fusion attention, and the SE module to improve the model’s generalization performance under varying working conditions. Existing studies have preliminarily demonstrated the application value of KAN networks in fault diagnosis. However, systematic research on the collaborative modeling of KAN with multi-scale convolutional feature extraction and attention enhancement mechanisms is still lacking. Therefore, to further exploit the advantages of KAN in complex nonlinear mapping, it is necessary to explore its deep integration with one-dimensional end-to-end diagnostic frameworks.

## 3. KanMSConv Method

### 3.1. KanMSConv Model Architecture

The overall architecture of the KanMSConv model consists of an input layer, a Stem module, three feature extraction stages, and an output layer, as illustrated in Figure 1. The input layer receives raw one-dimensional vibration signals, which are used to characterize the time-domain response information of bearings under different operating conditions. The Stem module is composed of a one-dimensional convolution layer, a batch normalization layer, a GELU activation function, and an average pooling layer. Its primary role is to perform preliminary encoding of the raw signal and to moderately compress the temporal dimension while preserving critical local information, thereby providing a more compact representation for subsequent deep feature learning.

Each stage consists of a multi-scale depthwise separable convolution module, an SE channel attention module, and a residual connection module. First, a 1×1 pointwise convolution is employed to perform channel mapping on the input features, so as to reduce the computational complexity of the subsequent multi-branch convolutions. Then, multiple depthwise convolution branches with different kernel sizes are used in parallel to extract multi-scale local features, thereby enhancing the network’s ability to model fault patterns under different receptive fields. Subsequently, the outputs of all branches are concatenated along the channel dimension, and feature fusion is achieved through normalization and nonlinear activation. This design helps simultaneously capture local impulsive components and multi-scale temporal variation information in vibration signals, thereby improving the richness and discriminability of feature representations.

After multi-scale feature fusion, the SE attention mechanism is introduced to perform adaptive recalibration of channel features. Through global information compression and channel weight learning, this module highlights key responses related to fault categories and suppresses interference from irrelevant or redundant information, thereby further enhancing feature representation capability. Meanwhile, to alleviate the gradient degradation problem that may occur during the training of deep networks and to improve feature reuse capability, a residual connection structure is incorporated into each stage. The output of the main branch is added element-wise to the input features adjusted by the shortcut branch, and the stage output is then obtained through GELU activation. This design not only ensures the stability of feature transmission, but also contributes to improving the convergence performance and robustness of the network.

As the network goes deeper, the model gradually completes the abstraction process from shallow local patterns to high-level discriminative features. Specifically, Stage1, Stage2, and Stage3 progressively expand the channel dimension of the feature maps while gradually compressing the temporal dimension, enabling the network to learn fault information at a higher semantic level. Finally, the output layer consists of a global average pooling layer and a KANLinear classifier. The global average pooling layer is used to compress high-dimensional temporal features into a compact global representation, while KANLinear is responsible for mapping the deep features into the target category space and producing the final fault identification results. Compared with traditional fully connected classification methods, the KAN classification head has certain advantages in modeling nonlinear decision boundaries, and can therefore further improve the model’s ability to distinguish complex fault states.

The proposed model performs preliminary encoding of the input signal through the Stem module, completes multi-scale feature learning, channel information enhancement, and residual feature fusion through the three-stage feature extraction process, and combines global pooling with the KAN classification head to achieve fault state identification. While maintaining relatively low computational complexity, the overall architecture balances feature extraction capability and classification discriminability, making it suitable for intelligent rolling bearing fault diagnosis tasks.

### 3.2. Multi-Scale Feature Extraction Module

**Figure 2:**
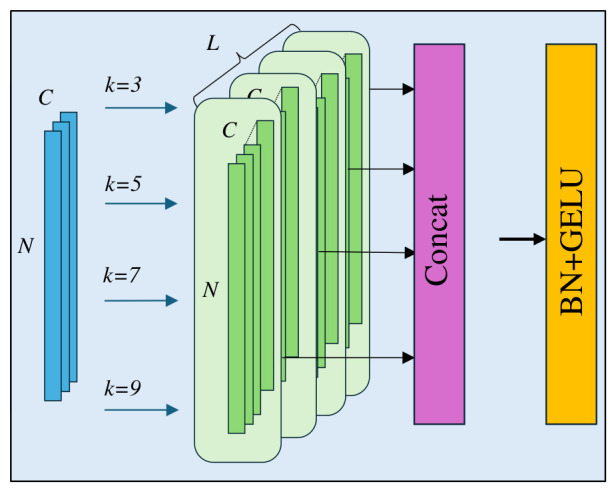
Multi-scale convolution.

As shown in Figure 2, multi-scale convolution is a feature extraction strategy that employs convolution kernels of different sizes in parallel. Its core objective is to simultaneously model short-range local patterns and long-range contextual dependencies within the same layer. Compared with single-scale convolution, which corresponds to only one fixed receptive field, the multi-scale design enables parallel filtering over multiple receptive fields to obtain multi-granularity representations. The outputs of different scale branches are then fused to form a richer feature representation.

To address the characteristics of one-dimensional fault signals, in which transient impulses, periodic components, and slow modulation coexist, the model adopts a parallel multi-scale depthwise convolution structure in the backbone of each stage. The core idea is as follows: first, a 1×1 convolution is used to perform channel remapping; then, depthwise branches with different kernel lengths are employed in parallel to extract patterns at multiple temporal scales; finally, the outputs are fused along the channel dimension, thereby achieving stronger time–frequency representation capability with relatively low parameter cost. The module input–output relationship is written as

(1) $$X\in R^{B\times C_{\mathrm{in}}\times N}\;\to \;Y\in R^{B\times C_{\mathrm{out}}\times N^{\prime }}$$

Multi-scale kernel set is defined as

(2) $$K=\left\{3,5,7,9\right\},\;M=|K|,\;C_{h}=\frac{C_{\mathrm{out}}}{M}$$

First, perform a 1×1 projection to obtain the shared latent space features.

(3) $$Z=\phi \left(\mathrm{BN}\left({\mathrm{Conv}}_{1\times 1}\left(X\right)\right)\right),\;Z\in R^{B\times C_{h}\times N}$$

where ϕ(·) denotes the GELU activation function. For each scale $k_{m}\in K$, depthwise convolution with $\mathrm{groups}=C_{h}$ is employed to perform parallel feature extraction:

(4) $$Y^{\left(m\right)}=\phi \;\left(\mathrm{BN}\;\left({\mathrm{DWConv}}_{k_{m},s}\left(Z\right)\right)\right),\;m=1,\ldots ,M$$

Its channel-by-channel, time-by-time position calculation can be written as:

(5) $$Y_{b,c,t}^{\left(m\right)}=\sum_{u=0}^{k_{m}-1}W_{c,u}^{\left(m\right)}\;Z_{b,c,t+u-p_{m}},\;p_{m}=\left\lfloor \frac{k_{m}}{2}\right\rfloor$$

The duration is determined by the convolution parameters:

(6) $$N^{\prime }=\left\lfloor \frac{N+2p_{m}-k_{m}}{s}\right\rfloor +1$$

Under odd kernel and same padding approximation, $N^{\prime }\approx N/s$. Concatenate the results of multiple branches along the channel dimension:

(7) $$Y=\mathrm{Concat}\left[Y^{\left(1\right)},Y^{\left(2\right)},\ldots ,Y^{\left(M\right)}\right],\;Y\in R^{B\times C_{\mathrm{out}}\times N^{\prime }}$$

### 3.3. SE Channel Attention Mechanism

**Figure 3:**
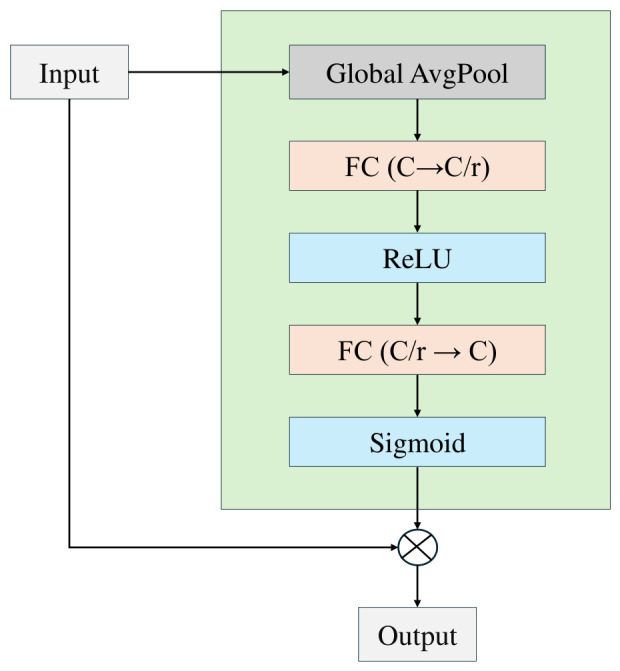
SE attention mechanism.

The basic structure of the SE attention mechanism is illustrated in Figure 3. First, global information aggregation is performed along the temporal dimension of the features (squeeze) to obtain a global descriptor for each channel. Then, a lightweight gating network is used to learn inter-channel dependencies and generate channel weights (excitation). Finally, the learned weights are applied to the original features, thereby enhancing informative channels and suppressing redundant ones.

Although the multi-scale feature extraction module can simultaneously capture short-term impulsive information and long-range modulation patterns, the contributions of different channels to fault discrimination are not identical. If all channels are directly fed into the subsequent classification head with equal importance, redundant responses may be introduced. This issue becomes more pronounced under strong noise interference or significant working condition variations, thereby weakening the model’s ability to focus on key fault patterns. Based on this consideration, a channel attention mechanism is introduced after the convolutional representation in each stage to process the features through a strategy of “statistics first, selection next, and recalibration finally” which can be formulated as:

(8) $${\hat{F}}_{b,c,t}=a_{b,c}\;F_{b,c,t}$$

The module first performs global aggregation in the time dimension, compressing the temporal response of each channel into a global descriptor. The significance of this is to transform local instantaneous changes into channel-level overall contributions, enabling the network to determine whether a channel is worth retaining at a higher semantic level. This process can be represented as:

(9) $$z_{b,c}=\frac{1}{N}\sum_{t=1}^{N}F_{b,c,t}$$

After obtaining the channel descriptors, a two-layer fully connected mapping with a bottleneck structure is employed to learn the channel weights. The process of compression–activation–restoration essentially models inter-channel dependencies in a lower-dimensional space. On the one hand, it reduces parameter overhead; on the other hand, it improves the separability between “important channels” and “less important channels” through nonlinear transformation. Finally, the Sigmoid function is used to constrain the weights to the range of 0∼1, thereby endowing them with a clear gating interpretation. After the weights are obtained, the module rescales the original features channel by channel to achieve adaptive recalibration:

From a functional perspective, this step is not simply a matter of “amplifying or shrinking numerical values”, but rather a process in which the network actively allocates more attention to frequency-band or morphological responses with greater diagnostic value. For example, channels that are more sensitive to impulsive faults can be enhanced, whereas channels that are more susceptible to background noise can be suppressed, thereby improving the discriminative stability across different samples and operating conditions.

### 3.4. KAN Network Module

**Figure 4:**
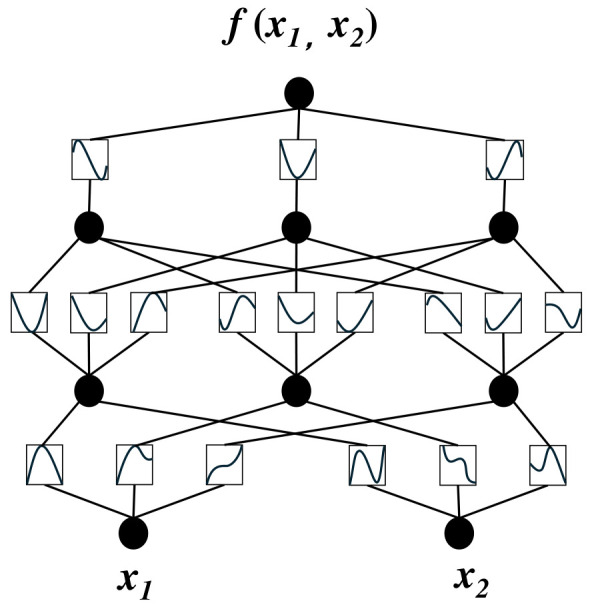
KAN network structure.

In traditional MLP classification heads, adjacent layers are connected through linear transformations Wx combined with the activation function σ(·). In contrast, the architecture of the KAN network, as illustrated in Figure 4, replaces the linear weights on the “edges” with learnable univariate functions, thereby decomposing high-dimensional mappings into a set of interpretable univariate nonlinear combinations. This design is more suitable for describing complex and nonlinear class decision boundaries. In this paper, KANLinear is introduced as the classification head on top of the global features produced by the convolutional backbone, in order to enhance the nonlinear fitting capability for fault patterns.

For a multivariate function f(x) with $x=(x_{1},\ldots ,x_{n})$, the mapping can be expressed as a composition in which an outer univariate function is applied to the summation of inner univariate functions:

(10) $$f\left(x\right)=\sum_{q=1}^{2n+1}\Phi_{q}\left(\sum_{p=1}^{n}\phi_{q,p}\left(x_{p}\right)\right).$$

here, $\Phi_{q}\left(\cdot \right)$ and $\phi_{q,p}\left(\cdot \right)$ are both univariate functions, reflecting the core idea of representing high-dimensional mappings through compositions of univariate functions.

After the backbone network completes multi-scale convolution and channel recalibration, the model first applies global average pooling to compress the temporal features into a global semantic vector, which is then fed into the KAN classification module to output class logits. Unlike the traditional fully connected layer that relies on “fixed linear weights,” the core idea of KAN is to model each connection as a learnable univariate function. From an expressive perspective, this means that each output category *j* is obtained by aggregating the nonlinear responses from all input dimensions *i*, rather than through a single linear weighting operation. Such functional connections are more suitable for handling the “nonlinear threshold effects” and “locally sensitive intervals” commonly observed in mechanical fault diagnosis: for the same feature dimension, the direction and magnitude of its contribution to different fault categories may vary significantly across different value ranges, and KAN can characterize such variations in a more refined manner. The main formulation is as follows:

(11) $$l_{j}=\sum_{i=1}^{d}\phi_{ji}\left(h_{i}\right)+b_{j}$$

where $\phi_{ji}\left(\cdot \right)$ is typically composed of a linear term and a spline basis function term, so as to improve the function fitting capability while maintaining trainability:

(12) $$\phi_{ji}\left(x\right)=a_{ji}x+\sum_{m=1}^{M}c_{jim}B_{m}\left(x\right)$$

Compared with a conventional linear layer, KANLinear changes the classifier from a global hyperplane-based mapping to an edge-function-based nonlinear mapping. A linear layer assigns one fixed scalar weight to each feature-class connection, whereas KANLinear learns a univariate function on each connection. Therefore, the contribution of one pooled feature can vary over different value intervals. This property is useful for bearing diagnosis because fault evidence is often interval-sensitive: impulse amplitude, modulation energy, and local frequency-band responses may support different fault classes only within specific ranges. The KAN layer is used only after global pooling, so the added nonlinear capacity is concentrated in the classifier while the convolutional backbone remains compact.

### 3.5. Loss Function

This paper uses the cross-entropy loss function as the optimization objective of the model to measure the difference between the model’s predictions and the true labels. Its expression is:

(13) $$L_{CE}=-\frac{1}{N}\sum_{i=1}^{N}\sum_{c=1}^{C}y_{ic}\log\left(p_{ic}\right),$$

where *N* denotes the number of samples, *C* denotes the number of classes, $y_{ic}$ represents the ground-truth label of the *i*-th sample for the *c*-th class, and $p_{ic}$ denotes the probability that the model predicts this sample to belong to the *c*-th class. The predicted probability is obtained by the Softmax function, namely,

(14) $$p_{ic}=\frac{\exp(z_{ic})}{\sum_{j=1}^{C}\exp\left(z_{ij}\right)},$$

where $z_{ic}$ denotes the output score of the model for the *c*-th class.

## 4. Experimental Results and Analysis

### 4.1. Model Settings

The model configuration is presented in Table 1, and the training hyperparameter settings are listed in Table 2. During the training stage, the proposed model employs the AdamW optimizer for parameter updating, with the initial learning rate set to $1\times 10^{-3}$ and the weight decay coefficient set to $1\times 10^{-4}$. The cross-entropy loss function is adopted as the optimization objective. To further improve the convergence stability of the model, a cosine annealing learning rate scheduling strategy is introduced to dynamically adjust the learning rate during the training process. The batch size is set to 32, and the total number of training epochs is set to 50. Meanwhile, the model parameters corresponding to the highest validation accuracy are saved and used as the final model for subsequent testing and performance evaluation.

### 4.2. Dataset Acquisition, Platform Roles, and Preprocessing Settings

The three datasets were obtained from different bearing test rigs. The platform information and sample descriptions are provided to clarify the physical sources of the public data rather than to indicate that the same hardware was used in all experiments. CWRU is used as a multi-load benchmark, PU provides a separate laboratory platform with artificial and real damages, and SDUST is used to evaluate recognition under different fault sizes.

For reproducibility, all input windows were converted into single-channel tensors and labels were assigned according to the folder or file names listed in Table 3. The exact analog-to-digital converter (ADC) bit depth is not available in the public files used for this study; therefore, ADC resolution was not treated as an experimental variable. The reproducible settings are the dataset source, selected channel or operating condition, sampling frequency when reported by the dataset provider, window length, stride, class mapping, and data split. For PU, source measurement files were assigned to training, validation, and test sets before window segmentation. Consequently, windows from the same source recording do not occur in different subsets.

### 4.3. Experimental Results and Analysis of the CWRU Dataset

**Figure 5:**
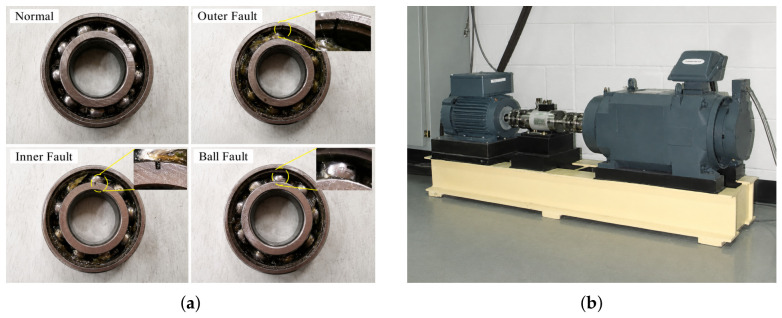
CWRU dataset includes bearing samples in different health states and the experimental testing platform [35]. (**a**) Bearing samples under different health states. (**b**) Experimental testing platform.

To validate the effectiveness of the proposed one-dimensional convolutional bearing fault diagnosis model integrated with KAN, this study adopts the rolling bearing vibration dataset provided by Case Western Reserve University (CWRU) [35] as the experimental data source. The corresponding CWRU experimental platform is presented in Figure 5. The commonly used sampling frequencies include 12 kHz and 48 kHz. The fault categories generally consist of inner race faults, outer race faults, and rolling element faults. Among them, outer race faults can be further distinguished according to the circumferential fault location on the outer race, such as at the 3 o’clock, 6 o’clock, and 12 o’clock positions. In addition, the dataset includes different motor load conditions, namely 0, 1, 2, and 3 hp.

In this study, vibration signals from the Drive End (DE) channel of the CWRU dataset are selected at a sampling frequency of 12 kHz. A ten-class task is constructed from the normal condition and combinations of fault type, size, and outer-race location. Experiments are conducted under four CWRU motor loads, namely 0 hp, 1 hp, 2 hp, and 3 hp. These load settings introduce variation in rotational speed, load path, signal amplitude, impulse intensity, and spectral energy distribution. The comparison therefore evaluates consistency across the available CWRU laboratory loads, rather than generalization to uncontrolled industrial operation.

Considering that the lengths of the raw data files are not identical, a sliding-window strategy is adopted to segment the continuous vibration signals in order to ensure consistent sample length. Specifically, each sample segment contains 1024 consecutive sampling points, and an overlap ratio of 0.5 is applied to increase the number of effective samples and improve training stability. After segmentation, the samples are labeled according to their categories and then fed into the proposed model for training and evaluation. The input dimension of the model is consistent with the above segmentation setting, i.e., a single-channel sequence with 1024 points. The proportions of the training set, validation set, and test set are set to 7:2:1. The experimental platform is configured as follows: the software environment includes PyTorch 2.5.1 and CUDA 12.1; the CPU is an Intel Core i7-14650H; and the GPU is an RTX 4060 Laptop (Intel Corporation, Santa Clara, CA, USA).

To compare benchmark performance with representative deep and conventional models, five deep-learning methods, namely VMD–CNN–Transformer [36], WDCNN [37], Deep ResNet [3], CWT-CNN [38], and AlexNet [39], are selected. An RBF-SVM [40] is trained on the raw flattened 1024-point windows using standardization, C=1, and γ=scale. It uses the CWRU training and test tensors described above, and precision, recall, and F1-score are macro-averaged.

As can be observed from Table 4, different models exhibit obvious differences in performance on the fault classification task. Overall, Deep ResNet and CWT-CNN show relatively moderate comprehensive performance, indicating that merely deepening the network through residual connections or relying on time–frequency feature transformation is still insufficient to fully capture the key discriminative information embedded in complex fault patterns. In contrast, AlexNet and WDCNN achieve better diagnostic performance, suggesting that convolutional architectures are highly suitable for vibration signal feature extraction. In particular, the wide-kernel design demonstrates certain advantages in capturing local impulsive features. Among the comparative models, VMD–CNN–Transformer delivers the best performance, indicating that the combination of signal decomposition, local feature extraction, and global dependency modeling can effectively enhance fault identification capability.

KanMSConv achieves the highest values across the reported CWRU metrics. The raw-window RBF-SVM obtains 67.43% accuracy, substantially below the deep models, indicating that the selected CWRU split is not solved equally well by a conventional classifier without learned feature extraction. However, the near-saturated results of several deep models indicate that this controlled benchmark alone provides limited resolution for comparing difficult-class behavior. The result supports effective representation learning on the evaluated CWRU split, rather than a general claim of industrial robustness.

**Figure 6:**
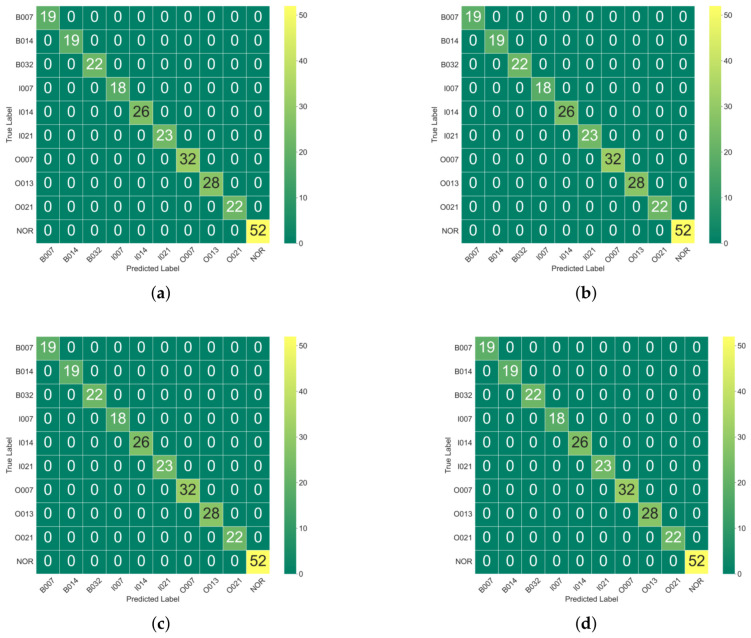
Confusion matrix results under different loads. (**a**) 0HP. (**b**) 1HP. (**c**) 2HP. (**d**) 3HP.

As shown in Figure 6, all categories are concentrated on the main diagonal under the four evaluated CWRU load conditions. This confirms consistent classification within these laboratory load settings. Because the test does not include external-machine interference, sensor drift, or unseen field equipment, it does not by itself establish generalization to uncontrolled industrial environments.

### 4.4. Experimental Results and Analysis on the PU Dataset

**Figure 7:**
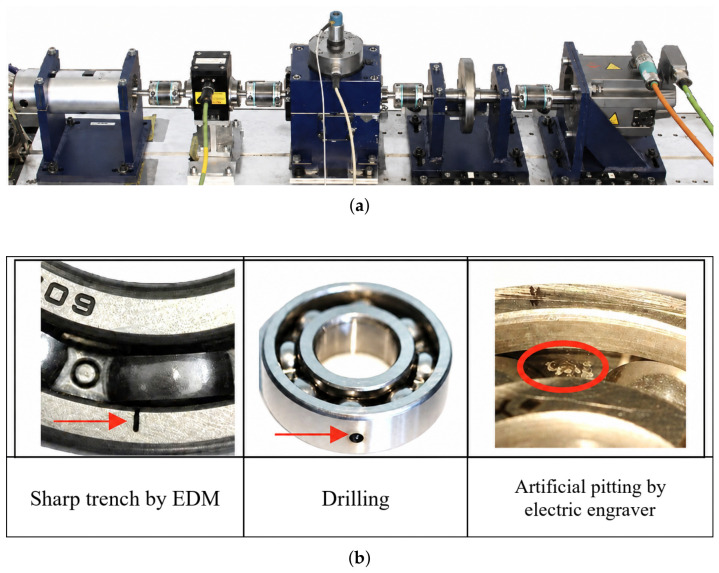
PU dataset experimental platform and schematic diagram of three artificial damage methods [10]. (**a**) Experimental platform. (**b**) Schematic diagram of three artificial damage methods.

To further validate the diagnostic capability of the proposed model, experiments are conducted on the bearing dataset from Paderborn University (PU) [10]. The PU dataset contains multiple bearing conditions, including both healthy bearing samples and artificially damaged bearing samples. The artificially induced faults include cracks and pitting. Cracks are generated by the electrical discharge machining (EDM) method to form regular cut-like fissures, whereas pitting is produced by drilling or electric engraving, as shown in Figure 7. Based on the differences in fault source and fault location in the PU dataset, a six-class fault diagnosis task is constructed, as listed in Table 5. Each sample contains 1024 consecutive points without overlap. To avoid distributing windows from one measurement recording across different subsets, the source files numbered 1–14, 15–18, and 19–20 for each class are assigned to the training, validation, and test sets, respectively, before segmentation. The PU experiment therefore evaluates classification across held-out source recordings under the selected N15_M07_F10 laboratory operating condition.

The PU comparison includes six published deep-learning methods and two conventional machine-learning baselines. The published methods are AMDC-CNN [41], MRSFN [42], Matrix-CNN [43], MCFCNN [44], CWT-RepLKNet [45], and SDP-CNN [46]. To directly test whether simpler classifiers are sufficient for the selected PU task, RBF-SVM [40] and random forest [47] are trained on the raw flattened 1024-point windows. The RBF-SVM uses standardization followed by the default RBF kernel with C=1 and γ=scale, and the random forest uses 200 trees with random seed 42.

Table 6 shows that the RBF-SVM and random forest reach 92.81% and 91.18% accuracy, respectively. Their relatively high scores confirm that the selected PU subset has favorable separability even for conventional raw-window classifiers. Nevertheless, KanMSConv exceeds the strongest conventional baseline by 7.12 percentage points in accuracy.

**Figure 8:**
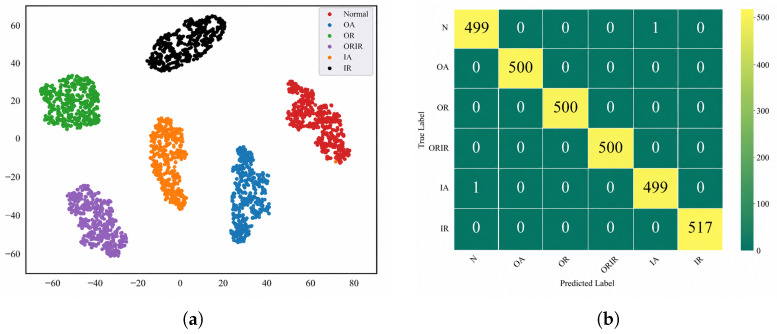
Feature visualization (t-SNE) and confusion matrix of six-class fault identification results from the PU dataset. (**a**) Feature visualization (t-SNE). (**b**) Confusion matrix.

Figure 8a visualizes the features produced by the trained KanMSConv backbone rather than the raw input windows. Therefore, the separated t-SNE clusters are an outcome of learned representation and nonlinear two-dimensional projection; they are not direct evidence that the original classes are intrinsically separable, nor are they a quantitative robustness test. The confusion matrix in Figure 8b confirms high accuracy on the held-out PU source files. Together with the conventional-baseline results, these observations indicate that the selected laboratory task is favorable while KanMSConv still provides a measurable benchmark improvement.

**Figure 9:**
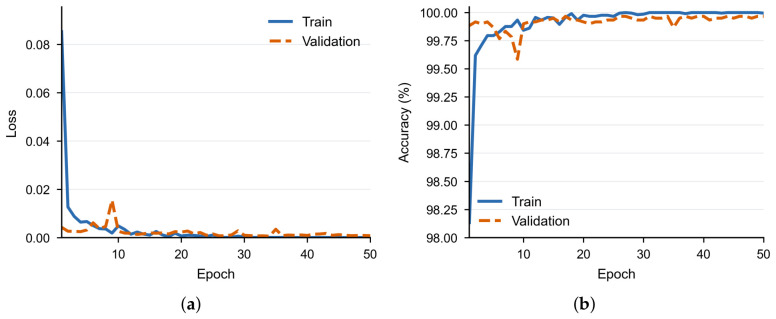
Training and validation curves on the PU dataset. (**a**) Loss curve. (**b**) Accuracy curve.

As shown in Figure 9, the training and validation curves converge rapidly and remain close under the selected PU split. This indicates no evident overfitting within the available laboratory recordings. It does not establish generalization to different machines, sensors, environmental noise, or operating regimes that are absent from the dataset.

### 4.5. Experimental Results and Analysis on the SDUST Dataset

**Figure 10:**
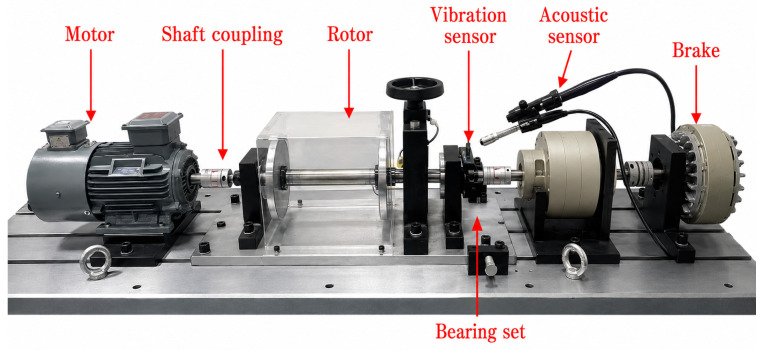
SDUST Dataset Testing Platform [48].

In this study, the SDUST bearing fault dataset [48] is introduced for experimental validation. The SDUST experimental platform is shown in Figure 10. This dataset includes normal operating conditions as well as multiple typical bearing fault states, and constructs multi-class samples by setting different fault sizes or severity levels, thereby enabling the evaluation of the model’s ability to distinguish different damage degrees.

To ensure consistency of operating conditions and reduce interference introduced by speed variation, all samples used for analysis are collected under the rotational speed condition of 1800 r/min. According to the dataset partitioning strategy of normal condition plus different fault types and fault sizes, a ten-class fault diagnosis task is constructed in this study. The SDUST samples are segmented into 2048-point windows without overlap, consistent with the experimental tensors. After segmentation, the samples are labeled according to their categories and then fed into the model for training and testing. In this way, the recognition accuracy and stability of the proposed model under different fault-size conditions can be systematically evaluated under a unified SDUST input length and consistent preprocessing settings.

Four models are selected for comparative experiments, including the traditional machine learning method SVM [40], as well as the deep learning models ResNet [49], WDCNN, and FCN-Desnet [50]. These methods cover technical routes ranging from a shallow raw-window classifier to typical convolutional neural networks and reveal performance differences on the SDUST fault-size classification task.

As shown in Table 7, SVM achieves 74.50% accuracy on the SDUST ten-class task, whereas the deep models achieve 97.08–99.80%. Compared with the highly separable PU task, this result provides complementary evidence from a benchmark in which a shallow raw-window classifier is substantially less competitive. Because SDUST is also collected on a controlled test rig, the comparison remains a laboratory benchmark rather than an industrial field test.

In contrast, ResNet, WDCNN, and FCN-Desnet all exhibit strong fault recognition capability, demonstrating the clear advantages of deep learning methods in vibration signal feature extraction and pattern discrimination. Among them, ResNet enhances feature learning ability through residual connections; WDCNN performs better in local feature extraction and classification stability; and FCN-Desnet also achieves strong overall performance, although there is still room for further improvement in terms of the balance of category prediction.

KanMSConv achieves the highest reported values on the SDUST benchmark. The large gap relative to SVM supports the value of learned representation for distinguishing fault-size classes in this dataset, while the smaller gaps among the deep models indicate that conclusions about architectural superiority should remain specific to the evaluated settings.

**Figure 11:**
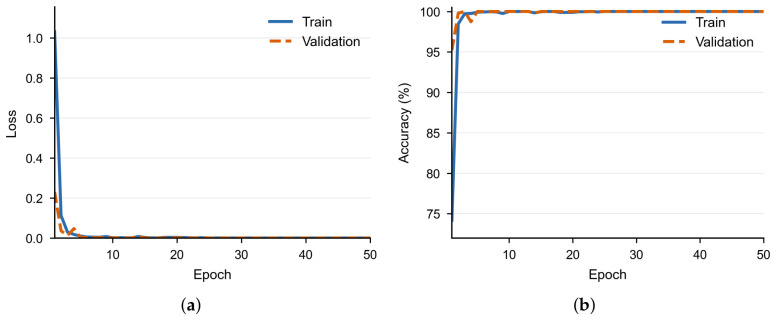
Training and validation curves on the SDUST dataset. (**a**) Loss curve. (**b**) Accuracy curve.

As shown in Figure 11, the model exhibits good convergence and stability during both the training and validation stages. The loss curves indicate that the loss value is relatively high at the beginning of training, but decreases rapidly within the first few epochs and quickly approaches zero. The validation loss also declines rapidly in a synchronized manner and remains fluctuating at a relatively low level during the later stage, indicating that the model is able to complete parameter optimization efficiently and learn stable feature representations.

### 4.6. Ablation and Computational Cost Analyses

To clarify the roles of the main modules, ablation tests compare the complete model with variants that remove MSConv or SE. Table 8 summarizes the ablation results on CWRU and SDUST. Because the two tasks are close to saturation, top-1 accuracy alone does not fully distinguish all variants. The results show that the multi-scale depthwise separable design preserves near-perfect accuracy with much fewer parameters than the ordinary convolution replacement, while the SE module reduces the test loss at the same accuracy level on SDUST. These results indicate that MSConv mainly contributes to parameter-efficient multi-scale feature extraction, whereas SE improves channel recalibration and prediction confidence.

Model-side CPU inference cost was assessed separately from end-to-end diagnostic latency. The timing test used batch size 32, random input tensors with the same length as each dataset, several warm-up iterations, and repeated forward passes. As shown in Table 9, the average per-sample forward time is less than 1 ms on CPU. This result supports low computational inference cost, but it does not measure end-to-end industrial latency or diagnostic robustness. Total latency would also include sensor acquisition, window buffering, signal transmission, and decision update time. For example, a 1024-point CWRU window at 12 kHz corresponds to about 85.3 ms of buffering before model inference.

### 4.7. Benchmark Separability and Industrial-Scope Limitations

All three datasets used in this study originate from controlled bearing test rigs. Their use supports reproducible comparison across selected loads, platforms, fault types, and fault sizes, but it does not reproduce the full variability of industrial condition monitoring. In particular, the experiments do not include interference from neighboring machines, sensor aging or remounting, long-term drift, severe and time-varying noise, transient operating regimes, or previously unseen equipment.

The results also show that benchmark difficulty differs across datasets. The high RBF-SVM and random-forest scores on PU confirm favorable separability for the selected six-class subset, whereas the lower SVM scores on CWRU and SDUST indicate more difficult raw-window classification tasks. Figure 8a should therefore be interpreted only as a visualization of the representation learned by KanMSConv. It does not establish that the original PU signals are visually separable or that the same clusters would be obtained for industrial field data.

Accordingly, the reported accuracy advantage is limited to the evaluated laboratory benchmarks. Practical deployment may require signal-quality monitoring, denoising, operating condition normalization, domain adaptation, or task-specific feature engineering. The effort needed for data acquisition, labeling, preprocessing, and model updating may be comparable to the effort required for classifier training.

## 5. Conclusions

This study develops a compact rolling bearing diagnostic model that integrates multi-scale feature extraction, SE channel recalibration, and a KAN classification head. The model achieves high accuracy on the evaluated CWRU, PU, and SDUST laboratory benchmarks while retaining a small parameter scale.

The RBF-SVM comparison across all three datasets shows that conventional raw-window classification performance varies substantially among benchmarks: 67.43% on CWRU, 92.81% on PU, and 74.50% on SDUST. The random forest also performs well on the selected PU task. KanMSConv retains a measurable accuracy advantage over these conventional classifiers, while the strong PU baselines confirm favorable separability for that selected six-class subset.

The computational analysis shows that the proposed model has a small parameter scale and sub-millisecond per-sample CPU inference time under the tested batch setting, which supports low-cost model-side inference after signal-window buffering. The ablation results also clarify that the MSConv block mainly improves compact multi-scale representation, while the SE module improves feature recalibration and prediction confidence. This computational result does not establish diagnostic validity in industrial environments. End-to-end industrial performance also depends on signal acquisition, buffering, transmission, hardware integration, interference, sensor stability, operating condition variation, and the representativeness of the training data.

Overall, KanMSConv provides a promising benchmark method for compact end-to-end bearing classification, but the present evidence does not support a general claim of superiority under real industrial conditions. Future work will prioritize external industrial validation, strong and time-varying noise, interference from adjacent machines, unseen equipment, cross-domain transfer, and the data-preparation requirements of long-term deployment.

## Figures and Tables

**Table 1:** Network configuration of KanMSConv.

Layers	Blocks	Parameters
Input signal	/	/
Input layer	Conv1D	c=32,k=15,s=2
	AvgPool1D	k=2,s=2
Stage 1	MSConv	$c=64,\;k_{i}=3,5,7,9,\;s=2$
	SE attention	r=8
	Residual projection	k=1,s=1
Stage 2	MSConv	$c=128,\;k_{i}=3,5,7,9,\;s=2$
	SE attention	r=8
	Residual projection	k=1,s=1
Stage 3	MSConv	$c=256,\;k_{i}=3,5,7,9,\;s=2$
	SE attention	r=8
	Residual projection	k=1,s=1
Output layer	Global average pooling	output size =1
	KANLinear	d=256,C=numberofclasses

**Table 2:** Optimization settings of KanMSConv.

Item	Setting
Optimizer	AdamW
Loss function	Cross-Entropy Loss
Initial learning rate	$1\times 10^{-3}$
Weight decay	$1\times 10^{-4}$
Learning rate scheduler	Cosine Annealing LR
Batch size	32
Epochs	50
Model selection	Best validation accuracy
Random seed	100

**Table 3:** Dataset acquisition sources and preprocessing settings used in this study.

Dataset	Platform and Signal Source	Selected Channel/Condition	Fault Classes	Window Setting
CWRU	Public motor-bearing test rig with accelerometers installed on the bearing housings; data were recorded by the CWRU acquisition system.	Drive-end vibration channel, 12 kHz sampling, 0–3 hp load conditions.	Ten classes: normal, ball faults, inner-race faults, and outer-race faults with three fault sizes/locations.	1024 points, stride 512, train/validation/test ratio 7:2:1.
PU	Paderborn bearing test rig including drive motor, torque shaft, bearing module, flywheel, and load motor; vibration was measured by the bearing housing accelerometer.	N15_M07_F10 operating condition, 64 kHz vibration signal.	Six selected bearing codes: K001, KA01, KA04, KB23, KI01, and KI14.	1024 points, no overlap; source files 1–14/15–18/19–20 assigned to train/validation/test.
SDUST	SDUST bearing fault test rig with an acceleration sensor installed close to the test bearing.	1800 r/min operating speed.	Ten classes: NC, IF0.2, IF0.4, IF0.6, OF0.2, OF0.4, OF0.6, RF0.2, RF0.4, and RF0.6.	2048 points, no overlap, train/validation/test ratio 7:2:1.

**Table 4:** Comparative results on the CWRU dataset.

Model	Accuracy	Precision	Recall	F1-Score
VMD–CNN–Transformer	$\underline{99.48\%}$	$\underline{99.51\%}$	$\underline{99.48\%}$	$\underline{99.10\%}$
WDCNN	96.02%	96.16%	96.08%	95.95%
Deep ResNet	93.15%	94.56%	93.13%	92.88%
CWT-CNN	94.06%	95.12%	94.00%	93.48%
AlexNet	95.82%	96.18%	95.82%	95.59%
RBF-SVM	67.43%	71.15%	62.10%	61.28%
**KanMSConv**	**100%**	**100%**	**100%**	**100%**

*Note:* Bold values indicate the best performance for each metric, and underlined values indicate the second-best performance.

**Table 5:** PU dataset six-class classification task label description.

Label	Class Code	Bearing Code	Fault Meaning in This Study
0	N	K001	Normal bearing condition
1	OA	KA01	Outer-race artificial damage
2	OR	KA04	Outer-race real damage
3	ORIR	KB23	Compound outer-race and inner-race damage
4	IA	KI01	Inner-race artificial damage
5	IR	KI14	Inner-race real damage

**Table 6:** Comparative results on the PU dataset.

Model	Accuracy	Precision	Recall	F1-Score
AMDC-CNN	87.08%	86.93%	86.56%	86.56%
MRSFN	91.78%	92.08%	92.13%	92.02%
Matrix-CNN	96.49%	96.69%	96.60%	96.60%
MCFCNN	95.21%	95.16%	95.03%	95.07%
CWT-RepLKNet	$\underline{98.80\%}$	97.95%	97.86%	97.90%
SDP-CNN	97.71%	$\underline{98.07\%}$	$\underline{98.10\%}$	$\underline{97.99\%}$
RBF-SVM	92.81%	94.50%	92.77%	92.55%
Random Forest	91.18%	92.50%	91.13%	90.32%
**KanMSConv**	**99.93%**	**99.93%**	**99.93%**	**99.93%**

*Note:* Bold values indicate the best performance for each metric, and underlined values indicate the second-best performance.

**Table 7:** Comparative experiment with four models.

Model	Accuracy	Precision	Recall	F1-Score
SVM	74.50%	74.74%	74.02%	74.31%
ResNet	97.08%	97.45%	97.10%	97.02%
WDCNN	$\underline{98.33\%}$	$\underline{98.40\%}$	$\underline{98.40\%}$	$\underline{98.40\%}$
FCN-Desnet	97.50%	97.37%	97.29%	95.27%
**KanMSConv**	**99.80%**	**99.81%**	**99.80%**	**99.81%**

*Note:* Bold values indicate the best performance for each metric, and underlined values indicate the second-best performance.

**Table 8:** Ablation results for the main KanMSConv modules.

Dataset	Variant	MSConv	SE	Accuracy	Test Loss
CWRU	KanMSConv	Yes	Yes	100.00%	0.0074
CWRU	w/o MSConv	No	Yes	99.21%	0.0388
CWRU	w/o SE	Yes	No	99.43%	0.0239
SDUST	KanMSConv	Yes	Yes	99.80%	0.0123
SDUST	w/o MSConv	No	Yes	99.45%	0.0233
SDUST	w/o SE	Yes	No	99.80%	0.0181

**Table 9:** Computational cost and inference-time measurement.

Dataset	Input Length	Classes	Parameters	Param. Memory	Batch-32 Time	Per-Sample Time
CWRU	1024	10	107,512	0.41 MB	24.02 ms	0.75 ms
PU	1024	6	97,272	0.37 MB	25.30 ms	0.79 ms
SDUST	2048	10	107,512	0.41 MB	27.10 ms	0.85 ms

## Data Availability

The datasets analyzed in this study are publicly available. The Case Western Reserve University bearing dataset is available from the Case Western Reserve University Bearing Data Center; the Paderborn University bearing dataset is available from Paderborn University; and the SDUST bearing dataset is available from the corresponding dataset source.
